# Treatment of Chronic Catarrh by Carbolic Acid without Trepanning

**Published:** 1883-01

**Authors:** John Lindsay

**Affiliations:** Huntington, L. I.


### IV.—TREATMENT OF CHRONIC CATARRH BY CARBOLIC ACID WITHOUT TREPANNING.

REPORTED BY JOHN LINDSAY, D.V.S.

July 5th, 1881, I was called to examine a horse at Clay Pits, Long Island. This animal was supposed to be suffering from glanders. As he was a valuable work horse the owner did not wish to destroy him without my advice.

When I arrived at the farm I was taken to a field where the animal was kept, which was half a mile from any road. The horse was a bad case to look at. He was discharging very offensive matter from both nostrils which had the odor of pus coming from a necrosed bone. The horse was much reduced in flesh and very weak. On examination I found him to be suffering from nasal catarrh, and on my stating this to the owner he wished me to try and cure him. The disease was of three years standing. At first I thought of trepanning, but having no instrument I concluded to try injecting the nostrils, knowing from experience that if I could reach the necrosed bones with my solution I could make a cure.

Mixing up ℥i. of Calvert's crystalized carbolic acid No. 2, to one pint of water, I injected two ounces into each nostril twice daily. After three days of this treatment there was a marked improvement, which after this was less pronounced, but there was a gradual and steady change for the better. At the end of two weeks the animal had improved much in general health, and at the end four months was entirely cured, and there has been no return of the trouble up to date.

V.—July 24th, 1882, I was called to see a horse suffering from a very offensive discharge from his nostrils of one year's duration. At times there was a marked subsidence of the discharge followed by acute excescerbations. When I saw the case it was in one ot the acute attacks. Upon examination I diagnosticated nasal catarrh.

I ordered the same treatment as used in the above case and in two months a cure was effected, with no recurrence.

VI.—August 10th, 1882, I was called to see a horse which could not breath easily and the owner feared the animal was developing heaves. The breathing was labored and there was marked evidence of obstruction in the nasal passages. There was not, however, the double action of the flanks commonly observed in horses. Upon inquiring I found that two months previous to my visit the horse had suffered with a severe discharge from the nostrils which had since ceased. But two weeks after the nasal discharge stopped he had trouble in breathing.

I came to the conclusion that the horse had been afflicted with chronic nasal catarrh and that the turbinated bones were plugged with thick pus. He was placed under the same treatment as the other two cases, and in three days began sneezing and blew from his nose two large masses of thick and cheesy pus, followed by a return of the discharge.

The continued use of the injections, however, terminated the case in a complete cure in one month.

HUNTINGTON, L. I.

